# Methods for quantifying methane emissions using unmanned aerial vehicles: a review

**DOI:** 10.1098/rsta.2020.0450

**Published:** 2021-11-15

**Authors:** Jacob T. Shaw, Adil Shah, Han Yong, Grant Allen

**Affiliations:** ^1^ Centre for Atmospheric Science, Department of Earth and Environmental Science, University of Manchester, Manchester, UK; ^2^ Laboratoire des Sciences du Climat et de l'Environnement (LSCE), CEA CNRS, UVSQ UPSACLAY, Gif sur Yvette, France

**Keywords:** methane, fluxes, UAVs, drones, emissions

## Abstract

Methane is an important greenhouse gas, emissions of which have vital consequences for global climate change. Understanding and quantifying the sources (and sinks) of atmospheric methane is integral for climate change mitigation and emission reduction strategies, such as those outlined in the 2015 UN Paris Agreement on Climate Change. There are ongoing international efforts to constrain the global methane budget, using a wide variety of measurement platforms across a range of spatial and temporal scales. The advancements in unmanned aerial vehicle (UAV) technology over the past decade have opened up a new avenue for methane emission quantification. UAVs can be uniquely equipped to monitor natural and anthropogenic emissions at local scales, displaying clear advantages in versatility and manoeuvrability relative to other platforms. Their use is not without challenge, however: further miniaturization of high-performance methane instrumentation is needed to fully use the benefits UAVs afford. Developments in the models used to simulate atmospheric transport and dispersion across small, local scales are also crucial to improved flux accuracy and precision. This paper aims to provide an overview of currently available UAV-based technologies and sampling methodologies which can be used to quantify methane emission fluxes at local scales.

This article is part of a discussion meeting issue 'Rising methane: is warming feeding warming? (part 1)'.

## 1. Introduction

Understanding and quantifying the global methane (CH_4_) budget is crucial for the prediction and mitigation of future climate change. Both emissions of CH_4_ to atmosphere and the absolute concentration of CH_4_ in the atmosphere have increased over the past decade [1], and there are concerns that climate feedbacks are further increasing emissions from natural sources [2]. Quantification of methane emissions at local scales (defined for the purposes of this review to be less than 1 km) is important for understanding natural methane production and emission processes, and also for informing emission reduction strategies and regulation for anthropogenic methane sources [2,3]. Emission flux quantification generally relies on either bottom-up, or top-down, methodologies, and validation of inventories requires a comparison of both approaches. Bottom-up inventories combine data-driven emission factors with statistical activity data, or use process-based modelling, whereas top-down methods combine atmospheric sampling with atmospheric advection (or dispersion) models to derive emission fluxes. Bottom-up estimates of the global methane budget (750 Tg yr^−1^ for 2017) are approximately 30% larger than equivalent top-down estimates (600 Tg yr^−1^ for 2017) [4]. There is therefore a need to reconcile and validate bottom-up emission inventory estimates and process-based models with top-down (measurement based) methodology.

Typically, quantifying methane emission fluxes requires the continuous sampling of atmospheric winds, coupled to absolute methane mole fraction measurements. Unfortunately, practical constraints typically require compromises to be made in both the temporal range and spatial resolution of measurements. Put simply, sampling methods cannot measure everywhere, all of the time. The empirical measurement data must typically be interpolated, assimilated or extrapolated, often by making use of models which simulate atmospheric dispersion and transport. All such flux quantification methods have intrinsic uncertainties and assumptions, many associated with the highly challenging simulation of atmospheric circulation, and assumptions therein.

A variety of platforms for the measurement of methane exist. These can range from ground-based systems (e.g. [5]), to instrumentation fitted onboard aircraft (e.g. [6–10]), to satellites, which monitor total atmospheric-column methane from low-Earth orbit (e.g. [11–13]). Measurement platforms can be geospatially fixed, in the form of towers (e.g. [14]) or long-term fixed-site monitoring stations (e.g. [15]), or geospatially flexible, in a moving vehicle for example (e.g. [16–18]). However, there remains a substantial sampling void between the ground, and altitudes of up to 100 m above ground, in which mobile platforms have been unable to operate until recently.

Large research aircraft lack the manoeuvrability to suitably sample methane emissions from small, local sources such as landfill or fossil fuel extraction facilities. Instead, large aircraft are more suited to regional-scale emissions quantification, such as those from wetlands or from cities, where they are not limited by their rapid speed or by flight restrictions (e.g. [19–21]). Lighter manned-aircraft have been used to measure fluxes from small, local sources, but typically require conditions including a stable background and an absence of significant extraneous sources (e.g. [22,23]). Such conditions are often found over the ocean, where manned-aircraft may also benefit from reduced flight restrictions compared with over land.

Unmanned (or uncrewed) aerial vehicles (UAVs; sometimes unmanned aerial systems or remotely piloted aerial systems), colloquially referred to as drones, offer a flexible monitoring platform for *in situ* atmospheric measurements or remote sensing of methane concentrations at the spatial scale of local sources (less than 1 km) and small facilities. UAVs can be equipped to dynamically monitor the lower atmosphere and planetary boundary layer (e.g. [24]) within the limits of local flight restrictions, and consequently their use and versatility in atmospheric measurement has expanded considerably in the past decade (e.g. [25–28]). The advent of small (but relatively imprecise) trace gas sensors has also reduced the cost of operating UAV monitoring platforms to near-consumable levels. UAVs are also uniquely placed for monitoring in hazardous, remote or difficult-to-access environments (e.g. [29,30]). Here, we review methods and approaches for quantifying methane emission fluxes which make use of UAVs as a measurement platform, focusing in particular on small UAVs with a maximum take-off weight of less than approximately 20 kg. UAVs below this mass are typically subject to reduced regulation; however, it should be noted that law and regulation varies considerably internationally. Regulatory standards will not be debated further in this review. We also do not review large remote-piloted UAVs (which are often used for military applications). Small UAVs are generally limited to operating at altitudes below 1 km and have shorter flight durations, making them ideally suited for trace gas detection in the near-surface atmospheric boundary layer [31,32]. The methods discussed within this review are most applicable to the characterization of methane emissions from localized- and facility-scale emission sources, be they natural or anthropogenic in nature.

## 2. Unmanned aerial vehicle platforms

**Figure 1.**
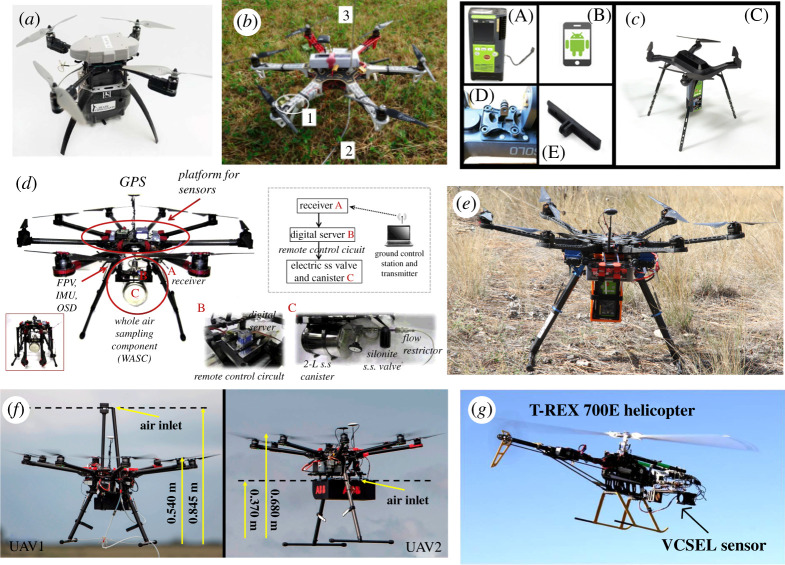
Selected examples showing UAV platforms and methane instrumentation from the literature. (*a*) The *Remote Methane Leak Detector* quadrotor UAV. Image from Yang *et al*. [33]. (*b*) A DJI F550 multicopter with installed sensors and tubing. Image from Brosy *et al*. [34]. (*c*) 3DR Solo quadrotor UAV and methane sensor. Image from Oberle *et al*. [35]. (*d*) Octorotor multicopter with a whole air sampling system and multiple environmental sensors attached. Image from Chang *et al*. [36]. (*e*) A hexacopter UAV with laser-based methane detector attached. Image from Emran *et al*. [37]. (*f*) Two adapted DJI Spreading Wings S1000 + octorotor multicopter UAVs—the left-hand UAV shows Teflon tubing to a ground-based instrument and the right-hand UAV has methane sensor attached. Image from Shah *et al*. [38]. (*g*) T-REX 700E robotic helicopter with methane sensor. Image from Khan *et al*. [39]. (Online version in colour.)

There are many different makes and types of small UAV platform available on the commercial market, with an expansive range of specifications, including weight, payload limit, maximum air speed, wing type and propeller type. The choice of UAV platform will not be discussed here in detail. Recent and comprehensive reviews of UAV platforms already exist (e.g. [31,32]). An example selection of UAV platforms previously used for methane detection and measurement is shown in figure 1. Small UAVs vary in mass on a scale from 100 g (micro-UAVs) through to 20 kg (and heavier), with implications for payload capacity and flight endurance. The UAVs can be powered by liquid fuel, but batteries are increasingly popular. Most small UAVs are limited to maximum air speeds of 15 m s^−1^, although this typically decreases during strong winds, and with increased UAV payload. Heavy payloads can also reduce flight endurance; small UAVs are typically limited to approximately 30 min of flight time, depending on sampling strategy, and the strength of the winds. Additional batteries (or liquid fuel) can be used to improve flight endurance, but only up to a threshold, where the weight of additional fuel becomes a burden. It should be noted that UAV platform specifications vary considerably, and that the values stated here are only representative of small UAVs at the time of writing. Technology continues to improve in regards to many of theseaspects.

Aside from classifications based on size and payload weight, UAVs can be broadly divided into fixed-wing and rotary-wing aircraft. Fixed-wing UAVs resemble traditional airplanes and the largest of these (e.g. NASA Global Hawk [31]) can benefit from enhanced stability (especially in more challenging weather conditions) and often an increased payload capability relative to rotary-wing UAVs [32]. However, small fixed-wing UAVs typically suffer from the same limitations as rotary-wing UAVs, in terms of stability and payload capacity. Small rotary-wing UAVs do have greater manoeuvrability and are able to perform vertical take-off and landing procedures, which offers practical advantages for fieldwork. Whereas small fixed-wing UAVs are typically faster and can sample across a greater spatial area in a shorter period of time. Both types of UAV have been used for trace gas detection and flux quantification. In summary, care must be taken when selecting a UAV platform, and consideration as to the exact application and intended operational environment should be made beforepurchase.

## 3. Wind measurement approaches

Accurate wind speed and wind direction is typically required for flux quantification. These can be measured (or inferred) from a suitable nearby monitoring station, equipped with anemometer instrumentation, but doing so may introduce some measure of uncertainty [40]. Preferably, the wind vector should be measured from onboard the UAV platform, and at a high spatial and temporal resolution, matching, if not exceeding, that of the methane measurement sampling rate. However, care must be taken to avoid interference of the wind field by the UAV platform itself. This is a particular challenge for rotary-wing UAVs, as the propellers constitute a barrier to ambient flow.

Various sensors for measuring wind velocity onboard a UAV already exist; multi-hole pressure sensors [41–43], or Pitot-tubes [44,45], are suited to fixed-wing UAVs. It is also possible to derive mean wind speed values in the absence of a designated sensor, through the use of a global navigation satellite system to infer wind speed from the ground speed and flight path bearing [46,47], using knowledge of the inertia of the UAV. Elston *et al*. [48] provide an overview of different types of wind sensors, and their applicability for fixed-wing platforms, and Rautenberg *et al*. [49] compare a number of different algorithms for the calculation of wind speed and wind direction against direct three-dimensional wind vector measurements. A five-hole probe mounted on a fixed-wing UAV has been observed to perform poorly for wind measurements when compared with measurements from a ground-based static sonic anemometer [40].

More recently, deriving wind measurements onboard rotary-wing UAVs has become possible [50]. This is traditionally considered more difficult due to strong perturbations in the wind flow induced by the rotating propellers, but results have been promising, with uncertainties below 0.5 m s^−1^ for wind speed, and 30° for wind direction [51]. Barbieri *et al*. [52] assessed 38 different UAV platforms carrying 23 unique two- and three-dimensional anemometers against reference measurements made from a meteorological tower and found that sonic anemometers mounted on rotary-wing UAVs provided the most accurate wind field measurements. However, while horizontal winds may be measurable using sonic anemometers on rotary-wing UAVs, three-dimensional (turbulence-scale) winds are far more problematic due to vertical flow disturbance by propellers. Mounting such sensors away from the plane of the propellers may overcome this, but the induced moment of adding mass away from the centre of gravity can significantly impact flight duration and lead to platform instability and oscillations.

## 4. Methane measurement approaches

A broad range of instrumentation for the measurement of methane exists, some of which may be more, or less, applicable for mounting onto a UAV platform [53,54]. Methane instrumentation applicable for small UAVs display a diverse variety of specifications, including measurement type, weight, measurement frequency, detection limit and precision (e.g. [55–58]). Some of these parameters, such as the instrument response time, will have an impact on the accuracy and spatio-temporal representability of measurements when using a mobile platform [59]. Instruments that offer excellent performance in terms of measurement sensitivity and precision have long been available, but these are generally more suitable for manned-aircraft (e.g. [7]), or ground-based applications (e.g. [15]), where there are fewer constraints on size, weight and power consumption (as well as cost).

UAV-based methane measurements have generally been made using three different methods, each with their own individual advantages and disadvantages [60].

**Figure 2.**
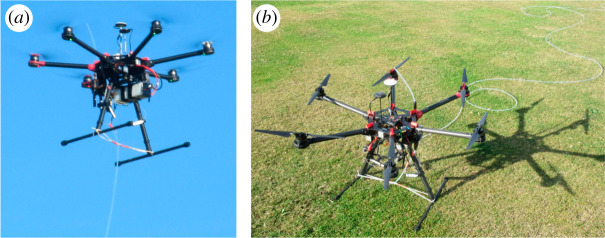
(*a*) A DJI Spreading Wings S900 UAV in flight; (*b*) The same UAV on the ground, with a 150 m long Teflon tether, connected to a methane instrument on the ground (not shown). Note that the tether is connected to an air inlet above the plane of the propellers. Image taken from Shah *et al*. [66]. (Online version in colour.)

1. *Air samples collected onboard the UAV.* Air samples are collected for later offline (laboratory-based) analysis of the methane mole fraction (e.g. [36,61–64]). This method can make use of the high-performance instrumentation (operated in the laboratory using the collected air samples) but usually results in discretized (snapshot), rather than continuous, spatially varying methane measurements. AirCore systems can be used for continuous atmospheric sampling but this typically results in low spatial resolution (greater than 20 m), even when sampling at air speeds below 2 m s^−1^ [63]. Additionally, offline analysis allows for the easy measurement of other variables, such as the ^13^C isotopic ratio of the methane, or other atmospheric trace gases (which may greatly aid source apportionment) assuming that chemical ageing of the sample has not taken place post-collection.
2. *Air sampled through tubing connected to the UAV.* Air is sampled through a tube (often referred to as a tether) carried by the UAV and measured by an instrument on the ground (e.g. [34,38,65] and figure 2). This method allows for the beneficial use of high-performance ground-based instrumentation which would otherwise be impossible for a UAV to carry, and which can also be operated continuously. Care must be taken to convolve the sampling time with the actual sampling location by accounting for the lag-time through the tubing, which can be on the order of 100 m in length. The use of a tether can also present logistical challenges, such as kinking of the tubing, or snaring on obstacles. A tether also limits a UAVs manoeuvrability and reduces both its horizontal and vertical range of motion and introduces flight hazards that may need to be mitigated.
3. *Air sampled live onboard the UAV.* This is perhaps the optimal method, but requires a sufficiently lightweight instrument, which can have consequences for both the precision of, and the resolution of, measurements. Onboard instrumentation allows the UAV full spatial freedom, but added weight may substantially impact flight duration. While lightweight but limited-performance methane sensors already exist (e.g. [67]), high-precision instrumentation less than 5 kg in weight are only just becoming commercially available. Rapid advances in the miniaturization of such instruments continue to be made, and instruments are known to be in development by various commercial manufacturers.

Many currently available lightweight methane sensors employ near-infrared laser sources. For example, Gardiner *et al*. [68] demonstrated a near-infrared tunable diode laser absorption spectrometer for methane detection (6 kg, ± 10% precision). Near-infrared sensors have been used to successfully map landfill methane hot spots from a rotary-wing UAV [69]. Mid-infrared laser sources make use of stronger methane absorption lines than near-infrared laser sources [55]. Golston *et al*. [70] presented a lightweight, mid-infrared methane sensor, with two designs weighing 1.6 and 4.6 kg, demonstrating 10 and 5 ppb precision at 1 Hz, respectively.

Non-dispersive infrared sensors have shown poor methane precision (±1160 ppb at 1 Hz) on UAVs, suggesting that they may be unable to quantify methane emission fluxes on the order of 1 g s^−1^ at local scales [67]. Non-dispersive sensors may have applications in the detection of large methane mole fraction enhancements (of at least 10 ppm) and should, like any new instrumentation, be rigorously characterized and tested for accuracy, precision and drift before use in scientific applications [67].

Open-path, tunable diode laser-based sensors can provide precise and fast measurements and are relatively lightweight [53,71]. Emran *et al*. [37] attached an open-path methane sensor (0.6 kg ± 10% precision), operating at 10 Hz, to their rotary-wing UAV. The same methane sensor was used to map the spatial distribution of methane over Arctic permafrost from a rotary-wing UAV, albeit with a methane concentration uncertainty of ±40% after calibration [35]. Nathan *et al*. [71] used a 3.1 kg open-path instrument to measure methane at 1 Hz, with a precision of approximately ±100 ppb. A miniaturized sensor (0.25 kg), based on technology built for the Mars Curiosity Rover, was used to detect controlled emissions of methane with ±10 ppb sensitivity at 1 Hz, but with potentially many hundreds ppb drift due to internal thermal changes [72]. Natural gas leaks were successfully detected using an open-path tunable laser diode absorption spectrometer attached to a fixed-wing UAV, with 90 min of flight time [73]. However, the instrument displayed substantial noise error in the field (up to 1.0 ppm), and there was only a 20% chance of successful leak detection, even when surveying large controlled emission rates [74]. An alternative open-path method used a dual-frequency-comb telescope on the ground, with an airborne retroreflector aboard a rotary-wing UAV, to measure methane, with a precision of ±6 ppb [75]. This method avoids the limitations resulting from mounting typically heavy instrumentation, while maintaining the advantages of flexible UAV-based spatial sampling [75].

Cavity-based laser absorption instrumentation typically offers higher precision measurements but tend to require more specialist operation and expertise than open-path technology. A low-power vertical cavity surface-emitting laser (2 kg) was tested aboard a robotic helicopter; results demonstrated a ±1% precision and also a long-term drift in methane measurements of around 1% [39]. Berman *et al*. [76] used a 19.5 kg off-axis integrated cavity output spectrometer with an 8 h battery life onboard a fixed-wing UAV to measure methane mole fraction in the Arctic. Laboratory testing demonstrated a precision of ±2.0 ppb (1*σ*) methane over an atmospherically relevant range of mole fractions, albeit with a small interference due to temperature [76]. Such a heavy payload will only be useful when using larger, sturdier UAVs with maximum take-off weights greater than 20 kg, however. A prototype of a miniaturized off-axis integrated cavity output spectrometer (3.4 kg, 32 W, 2 ppb at 1 Hz precision) built specifically for UAV use was tested on a rotary-wing UAV and successfully used to calculate methane fluxes [38,65]. Similarly, an open-path cavity ring-down spectroscopy instrument (4.1 kg) was successfully integrated onboard a rotary-wing UAV, demonstrating in-flight precisions of between 10 and 30 ppb [77]. This platform was used to monitor isolated plumes from controlled releases of methane of 0.5 g s^−1^ [77]. Elsewhere, a ground-based cavity ring-down spectrometer was tethered to a rotary-wing UAV, providing stable methane measurements with a precision of ±7 ppb [34]. The 50 m length of tubing, attached to an inlet 30 cm above the UAV propellers, gave an additional payload weight of just 0.65 kg. Results showed good agreement between the UAV-based and adjacent tower-based methane mole fraction measurements [34].

Additionally, methane emissions can be detected qualitatively using thermal imaging. This is particularly useful for leak detection, but not for flux quantification, without additional supporting measurements. Tratt *et al*. [78] used airborne thermal-infrared hyperspectral imaging spectrometry to visualize methane emissions from various fossil fuel sources while Lehmann *et al*. [79] used high-resolution colour infrared images to map methane hot spots and upscale independent chamber-derived methane emission fluxes in a natural peat bog ecosystem. Tanda *et al*. [80] used airborne infrared thermography to detect methane emissions from landfill and were able to provide a crude estimate of methane flux rate using a simple relationship between observed heat exchange and the heat associated with methane production. Similarly, a UAV-mounted thermal-infrared camera was used to visualize methane emissions from a Danish landfill, but methane flux was quantified more rigorously using independent surface chamber measurements [81]. Three different imaging sensors (RGB, thermal-infrared and near-infrared), mounted on a rotary-wing UAV, were compared for their applicability for mapping landfill emissions in Lithuania [82].

Non-optical approaches have also been tested for UAV-mounted applicability. Solid-state, or chemical, sensors offer a low-cost, low-weight alternative to typically bulky and technologically expensive laser-based systems (e.g. [83,84]). Taguem *et al*. [85] tested a series of metal oxide sensors in the laboratory for the purpose of UAV-mounted monitoring, but did not report any operational flight data. Despite reporting a linear response between sensor readings and methane concentration, Taguem *et al*. [85] concluded that in-field sampling may be difficult due to low sensitivity to methane and sensor response to other trace gases. Ali *et al*. [86] used a semiconductor-based methane sensor for their UAV-based measurements, albeit with a high limit of detection (10 ppm) which may only be suitable for sampling methane very close to emission sources.

A summary of the methane instrumentation that has been deployed on UAV platforms is presented in table 1.

**Table 1.** Summary of methane instrumentation deployed on UAV platforms.

measurement type	mass (kg)	power consumption (W)	precision	resolution (Hz)	notes	reference
off-axis integrated cavity output spectroscopy	19.5 (with ancillary systems)	70	2 ppb (in laboratory)	1	temperature interference	[76]
mid-infrared open-path wavelength modulated spectroscopy	4.6 1.6	30	5 ppb 10 ppb	1–10		[70,87]
handheld open-path	0.6		10%	10	path-averaged concentration	[35,37]
near-infrared standoff tunable diode laser absorption spectroscopy	1.4	1			path-averaged concentration	[69]
custom open-path	3.1	25	100 ppb or 10%	1	path-averaged concentration	[71]
near-infrared vertical cavity surface-emitting laser	2	2	1%	1	long-term drift around 1%	[39]
near-infrared tunable diode laser absorption spectroscopy	6		10%	0.5		[68]
tunable laser spectroscopy	0.25		10 ppb	1	potentially 100 s ppb drift due to thermal interference	[72]
open-path cavity ring-down spectroscopy	4.1	12	5–10 ppb (in laboratory)	1		[77]
			10–30 ppb (in field)			
open-path tunable laser diode spectroscopy					noise on the order of 1000 ppb	[73,74]
					path-averaged concentration	
non-dispersive infrared	1.5 (with ancillary systems)	4.2	1160 ppb	1		[67]
dual-frequency-comb telescope (ground-based)	N/A		16 ppb	0.1	UAV carried an airborne retroreflector	[75]
					path-averaged concentration	
off-axis integrated cavity output spectroscopy (tethered)	N/A	35	0.7 ppb @ 1 Hz	10		[38,65]
off-axis integrated cavity output spectroscopy	3.4	32	2 ppb @ 1 Hz	5		[38,65]
cavity ring-down spectroscopy (tethered)	N/A		7 ppb			[34]

## 5. Methane flux quantification

A variety of methods have been used to quantify methane emission fluxes from many emission sources. Here, we review many of the techniques that have been applied to UAV-based measurements.

### (a) Mass balance box modelling

Mass balance (or mass budget) box methods consider the conservation of the mass of methane within a system (or volume), usually conceptualized as a box. This method has been routinely used in manned-aircraft-based flux quantification, for deriving methane emission fluxes from cities (e.g. [20,88]), from wetlands [19] and from industrial regions (e.g. [6,21]). The emission flux is typically quantified as demonstrated by equation (5.1):

5.1 $$F=\int_{z_{1}}^{z_{2}}\int_{x_{1}}^{x_{2}}([CH_{4}]-{\left[CH_{4}\right]}_{b})U_{⊥}\text{d}x\text{d}z,$$

where *F* is the emission flux, and the enhancement of methane is quantified by subtracting the ‘background’ methane concentration ([*CH*_4_]*_b_*; usually measured upwind of the source) from measurements of methane within the emission plume ([*CH*_4_]) [89]. *U*_⊥_ is the wind speed perpendicular to the vertical plane upon which the plume is projected. These values are integrated over both the horizontal extent (between *x*_1_ and *x*_2_), and the vertical extent (between *z*_1_ and *z*_2_), of the emission plume. It should be noted that in order to derive a flux in units of mass per unit time (g s^−1^ for example), many direct methane concentration measurements will need to be converted from the mole fraction (in ppb or ppm) typically recorded by many instruments, to a mass density (in g m^−3^ for example).

The mass balance method generally requires that the wind field is constant between upwind and downwind measurements, i.e. that the wind speed and wind direction does not change. However, wind field variability can be implicitly accounted for through robust uncertainty propagation [8]. When using manned-aircraft for sampling, the atmosphere is usually considered to be vertically well-mixed, and this may be the case if measurements are made sufficiently downwind of the emission source. If this cannot be assumed, then the vertical variability in methane must be either measured, approximated or both [90]. For UAV-based sampling, where measurements can be made much closer to the source, the plume is unlikely to be vertically well-mixed, but dense spatial sampling is still important for flux accuracy [71].

**Figure 3.**
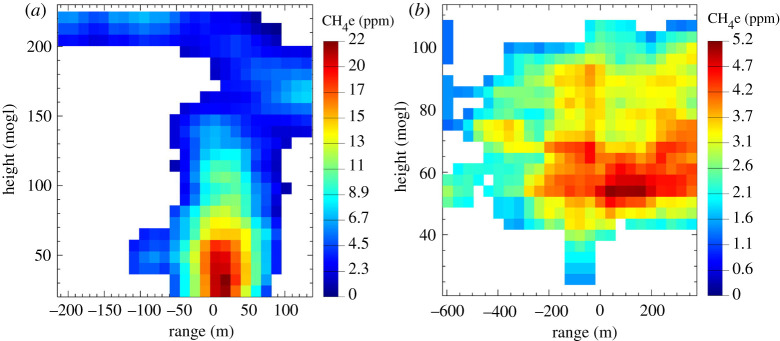
Methane enhancement (CH_4_e) over background, interpolated onto a two-dimensional flux plane using emission correlations between CO_2_ measured from a fixed-wing UAV platform and methane at a landfill site in the UK on: (*a*) 27 November 2014; and (*b*) 5 March 2015. Figure taken from Allen *et al*. [40]. (Online version in colour.)

Allen *et al*. [40] used the mass balance method, in conjunction with UAV-based measurements of CH_4_ and CO_2_ to quantify a methane flux from landfill. They used two UAV platforms: a fixed-wing UAV equipped with a high-precision CO_2_ instrument (non-dispersive infrared, 0.3 kg, 1% precision at 1 Hz sampling rate) and a rotary-wing UAV tethered to an off-axis Integrated Cavity Output Spectrometer (ICOS; Los Gatos Research (now ABB) Ultraportable Greenhouse Gas Analyzer [91]). The rotary-wing UAV was used to determine the vertical mixing of methane and CO_2_, while the fixed-wing UAV was used for horizontal measurements of CO_2_, from which the horizontal distribution of methane was inferred using emission ratios characteristic of landfills (figure 3). Methane fluxes were quantified for two flights (0.14 ± 0.09 and 0.05 ± 0.03 kg s^−1^) with the uncertainties mainly influenced by the variability in the methane background, and the variability in the wind. Their reported uncertainties were comparable to uncertainties derived from other non-UAV-based landfill flux estimation methods (e.g. [92]). However, CO_2_ can be a poor proxy for landfill methane emissions in the presence of interfering (off-site) sources [60] and the use of a dedicated methane instrument on both UAVs would have been preferable [40].

Nathan *et al*. [71] used the mass balance method to estimate the methane emission rate from a compressor station. They deployed an open-path methane sensor (3.1 kg, 10 Hz, 100 ppb precision) on a fixed-wing UAV. Kriging (e.g. [20,93]) was used to interpolate between the spatially sparse measurements on the two-dimensional vertical sampling plane. The mean methane emission flux from 22 flights was estimated to be 14 ± 8 g s^−1^. This was larger than the fluxes quantified by two additional, non-UAV-based methods, which yielded methane emission rates of 5.8 g s^−1^. Nathan *et al*. [71] noted that their method was limited by the low density of their spatial sampling and found potential biases resulting from the emission plume centre changing over time. The variability in the plume morphology, distinguished as an instantaneous plume as opposed to a time-averaged plume, means that there are problems with using interpolation methods (such as kriging) to infer the spatial distribution of the methane plume [60].

**Figure 4.**
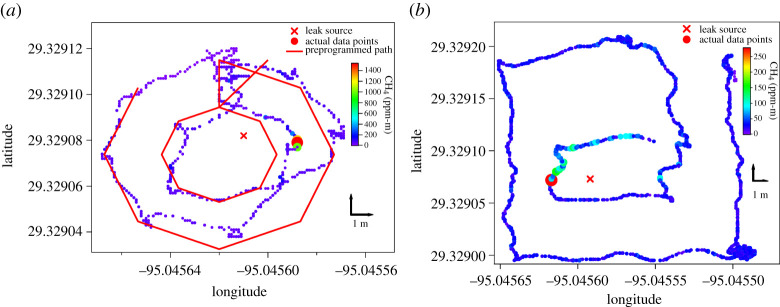
Examples of concentric UAV flight paths are used for estimating the emission rate of target infrastructure via path-integrated methane measurements and the mass balance method [33]. Crosses indicate the location of the leak source, and the colour and size of the data points represent the path-integrated CH_4_ mixing ratio (ppm-m): (*a*) demonstrates a concentric octagonal flight path and (*b*) demonstrates a concentric rectangular flight path. Figure from Yang *et al*. [33]. (Online version in colour.)

A variant of the mass balance method was used to detect and quantify natural gas leaks using a UAV equipped with a backscatter tunable diode laser absorption spectrometer [33]. This instrument measures path-integrated methane with a sensitivity of 5 ppm-m, equivalent to a 500–5000 ppb methane precision at a near-field distance of 1–10 m. As open-path methane instruments directly measure the total vertical concentration of methane, this removes the need to integrate over the vertical extent of the plume ($\int_{z_{1}}^{z_{2}}\left([CH_{4}]-{\left[CH_{4}\right]}_{b}\right)\;\text{d}z$). The methane emission flux was estimated from the mean of multiple mass balances performed on concentric paths flown around the target infrastructure at different distances (figure 4). The method tended to underestimate emission fluxes, particularly when the wind speed was low and wind direction variable. Estimated fluxes under the highest wind speeds and steadiest wind direction were approximately 50% accurate. Uncertainty in the GPS location data used in their study also reduced reliability [33].

The mass balance approach, incorporated alongside algorithms to both detect and localize leaks, was used to quantify natural gas emissions using a UAV-based remote sensing measurement platform [87]. Methane measurements were made using an open-path sensor, and quantified fluxes were consistent with those derived using the alternative methodology.

### (b) Gaussian plume inversion

Gaussian plume models can be used to model enhancements in methane concentration downwind of a point source using Gaussian statistics [94]. This method has been used for quantifying methane emissions from oil and gas infrastructure using ship-based measurements (e.g. [95,96]), van-based mobile measurements (e.g. [97,98]), and from stationary-site measurements (e.g. [99,100]). The methane flux from a single-point source can be quantified by inverting equation (5.2), which models the downwind methane enhancement as dependant on the methane background, the emission flux rate, the dispersion rate and the wind speed [94].

5.2 $$\begin{array}{r} {}[CH_{4}](y,z)=\left(\frac{F}{2\pi U_{⊥}\sigma_{y}\sigma_{z}}\times \exp\left(\frac{-y^{2}}{2{\sigma_{y}}^{2}}\right)\times \left(\exp\left(\frac{-{\left(z-H\right)}^{2}}{2{\sigma_{z}}^{2}}\right)+\exp\left(\frac{-{\left(z+H\right)}^{2}}{2{\sigma_{z}}^{2}}\right)\right)\right)+{\left[CH_{4}\right]}_{b}, \end{array}$$

where *F* is the emission flux, *U*_⊥_ is the perpendicular wind speed, *H* is the height of the emission plume source above the ground and *σ_y_* and *σ_z_* are Gaussian dispersion parameters of the plume in the *y* (across plume, perpendicular to wind direction) and *z* (vertical) directions, respectively. It should be noted that for the Gaussian plume method, *y* is conventionally used to refer to the cross-plume coordinate, whereas *x* is used to denote the same quantity for the mass balance box method.

Nathan *et al*. [71] used the Gaussian plume method (in conjunction with UAV-based sampling) to compare against methane fluxes derived using the mass balance method. The Gaussian plume method converged to a methane flux of 23 g s^−1^, larger than both the mass balance derived flux (14 ± 8 g s^−1^), and the two independent non-UAV-derived fluxes (both 5.8 g s^−1^). Ali *et al*. [86] used the Gaussian plume method and a UAV platform to quantify landfill methane fluxes but were limited by the poor sensitivity of their methane sensor.

The Gaussian plume method usually requires a large amount of time averaging in order for the instantaneous plumes to resolve into an observed Gaussian plume morphology suited to Gaussian inversion. When sampling in close proximity to an emission source (less than approximately 100 m), the time scale of measurements is unlikely to allow for adequate time averaging, and a non-Gaussian distribution of methane may be observed. The turbulent fluctuation of the wind field combined with the short flight duration of many UAVs may result in the measurement of an instantaneous plume and, therefore, a poorly defined plume morphology. However, repeated flights (or longer duration sampling) in less variable wind conditions, combined with carefully designed flight patterns can mitigate this. Individual case studies would need to be evaluated for the conditions specific to the environment at the time of sampling, by comparing data with fitted Gaussian plume assumptions.

A recently developed adaptation of the Gaussian plume flux inversion, referred to as the near-field Gaussian plume inversion (NGI) technique, was adapted for downwind sampling of turbulent plumes close to the emission source [66]. In the NGI method, the methane flux density is calculated at all points across a vertical plane perpendicular to the mean wind direction, using a method similar to that used in the mass balance approach. An emission flux estimate is then derived by fitting the measured methane flux density values to a modelled (Gaussian) flux density, using an adaptation of the two-dimensional Gaussian plume model (equation (2.2)), with the exclusion of wind speed, which is implicitly and spatially accounted for when deriving flux density. A final (optimally fitted) methane emission flux is then calculated by iteratively solving four simultaneous equations, under the assumption that *σ_y_* and *σ_z_* increase linearly with distance from the source location (suitable over small distances). This method requires adequate spatial sampling density in both the *y* and *z* directions to resolve a flux [66].

Shah *et al*. [38] tested the NGI method using controlled releases of methane. Two rotary-wing UAVs were used: one tethered to a ground-based off-axis ICOS instrument (Los Gatos Research (now ABB) Micro-portable Greenhouse Gas Analyzer), and the second carrying a miniaturized, lighter prototype of the same instrument. The approach was tested for 22 flight surveys, of which 19 produced results with good agreement with the known controlled emission flux. The reported lower (17 ± 10%) and upper (227 ± 98%) uncertainty bounds resulted from the variability in the position of instantaneously observed plumes and spatially sparse sampling. While these uncertainties are large, Shah *et al*. [38] concluded that the NGI method is viable in situations where access to an emission source site is prohibited or impractical.

**Figure 5.**
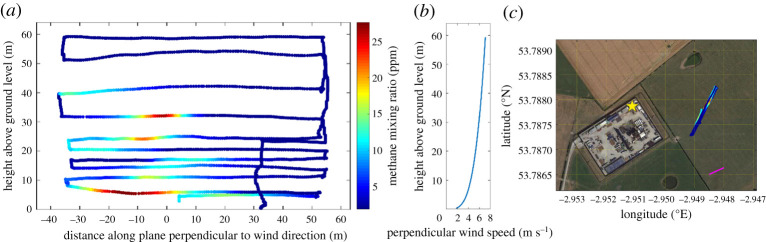
(*a*) Methane mixing ratios (ppm) measured along a UAV flight track showing an example of a geospatially mapped methane plume. Measurements were made on 14 January 2019 at a hydraulic fracturing facility in the UK (see Shah *et al*. [100] for details); (*b*) Perpendicular wind speed measured during sampling using a two-dimensional sonic anemometer mounted on the UAV platform. (*c*) Location of sampling (Google Maps © dated 26 September 2018) showing the UAV flight track. The arrow (bottom right corner) represents the mean wind direction (254.9°) during sampling. Figure taken from Shaw *et al*. [100]. (Online version in colour.)

The NGI method was used to quantity the methane emission flux resulting from unintended cold-venting during flowback operations at a hydraulic fracturing facility in the UK [65] (figure 5). In this instance, two UAV platforms were used for methane measurements: a rotary-wing UAV, tethered to a ground-based off-axis ICOS instrument (Los Gatos Research (now ABB) Micro-portable Greenhouse Gas Analyzer) and a second rotary-wing UAV, carrying a miniaturized prototype of the same instrument intended for UAV-based use. Calculated methane fluxes were between 9 and 156 g s^−1^, in good agreement with fluxes derived from nearby ground-based monitoring [100]. The range of fluxes derived using the NGI method therefore likely reflects the time-varying nature of the emission source, as well as the derived uncertainty [65].

Another approach related to the Gaussian inversion method was used by Golston *et al*. [87] to quantify methane fluxes from natural gas stations. The method was based on the inverse relationship between methane enhancement and wind speed, and the linear relationship between methane enhancement and emission flow rate (see equation (5.2)), derived from a set of test data. Dispersion in the *z* direction (*σ_z_*) was accounted for by using an open-path methane sensor measuring column-integrated methane, and dispersion in the *y* direction (*σ_y_*) was assumed to be small, due to the close proximity to the source. Quantified fluxes were shown to be in agreement with the mass balance approach used in the same study [87].

### (c) Alternative approaches

A methane emission flux could feasibly be quantified using UAVs in conjunction with the tracer release method, and a controlled release of a reference tracer gas at a known rate from the source origin (e.g. [92,101–103]). This method removes the problematic need for both meteorological data and atmospheric modelling of transport and dispersion, but requires instrumentation capable of measuring the tracer gas at high precision in addition to high-precision methane measurements. The tracer release approach relies on the assumption that the co-emitted tracer gas will disperse in the atmosphere in an identical way to that of the methane. In this way, the emission flux of methane can be quantified using the known emission rate of the tracer gas, and the ratio of the tracer gas to methane concentration (above background). To the best of the authors' knowledge, direct use of this method in conjunction with a UAV platform has not yet been made.

Alternative atmospheric inversion approaches could also be applied to UAV-based measurement data to quantify methane fluxes. For example, Lagrangian particle dispersion models simulate the paths of many massless particles as they travel with the local wind field (e.g. [104]) and have been used to quantify methane fluxes (e.g. [100,105]). Lagrangian particle models may be more physically valid than Gaussian inversion approaches, which tend not to model turbulence in the wind, but are computationally more expensive.

Ravikumar *et al*. [106] compared the methane flux quantification capacity of several vehicle, UAV and plane-based technologies but did not present their flux methodology. The accuracy of UAV-based flux quantification, as well as the ratio of positive to negative identification of leaks, was found to be comparable to vehicle-based methods. Ravikumar *et al*. [106] concluded that UAV technologies are still in their infancy, require large amounts of labour and suffer from regular battery changes and frequent groundings during adverse conditions. Despite this, their capability for quantifying emissions from tall infrastructure, and during calmer atmospheric conditions, was noted as being particularly advantageous relative to vehicle-based monitoring.

## 6. Conclusion

The literature reviewed here demonstrates the clear capability of small UAV-based measurement platforms for quantifying methane fluxes from a range of sources at local scales (less than 1 km). Such capability has only become available over the past decade, as a result of advances in both UAV technology and the miniaturization of instrumentation for the measurement of methane. Lightweight and high-performance instrumentation, with the capacity to measure methane with a precision on the order of a few ppb, are now becoming commercially available (at the time of writing), and developments continue to be made at pace. Such instrumentation will enable more accurate flux quantification, particularly of smaller emission sources which may not be captured by current methodology. Further developments in UAV technology would also be highly beneficial. Enhanced flight duration, as a result of improved battery capacity, would be extremely advantageous in terms of plume mapping, allowing for extended temporal ranges and refined spatial resolution of methane measurements. A summary of advantages and disadvantages of different UAV platform types, sampling methodologies, and flux quantification techniques is presented in table 2.

**Table 2.** Summary of advantages and disadvantages for different UAV platforms, sampling methods and flux quantification techniques.

UAV wing type	advantages	disadvantages
fixed-wing UAV	— greater flight speed so can cover a larger spatial area	— require large and open areas free of obstacles for take-off and landing — may require additional apparatus for take-off (e.g. catapult)
rotary-wing UAV	— greater manoeuvrability — vertical take-off and landing capability — easier to control — can hover at a fixed position	— wind speed and wind direction measurements can be more challenging owing to propeller air-flow — adding mass (e.g. instrumentation) away from the centre of gravity can lead to poor flight stability

sampling method	advantages	disadvantages
air samples collected onboard the UAV	— can use high-precision instrumentation for offline analysis irrespective of mass, size, cost etc. — can measure other variables such as ^13^C isotopic ratio of methane, or other atmospheric pollutants which can aid source apportionment — full spatial freedom for the UAV platform	— usually results in discretized (snapshot) sampling as opposed to continuous sampling
air sampled through tubing connected to the UAV	— continuous sampling — can use high-precision ground-based instrumentation irrespective of mass, size, cost etc. — could measure other atmospheric variables (e.g. ^13^C, or other air pollutant)	— must account for the lag-time of the air through the tubing to convolve sampling time with measurement location — limits the range and manoeuvrability of the UAV — the tubing can present physical and logistical challenges, such as snaring, or kinking — weight of tubing increases with sampling height (as more tubing is lifted) reducing flight duration
air sampled live onboard the UAV	— continuous sampling — full spatial freedom for the UAV	— added weight reduces potential flight duration — instrumentation balance between size, weight, cost versus precision, measurement frequency etc.

flux quantification technique*	advantages	disadvantages
mass balance box model	— can be adapted for either point measurements, or path measurements — can be used for multiple point emission sources, or dispersive emission sources (such as landfill)	— requires dense spatial sampling or some form of interpolation/extrapolation — assumes steady winds
Gaussian plume inversion	— can be adapted for either point measurements, or path measurements	— assumes Gaussian distributed plume (time-averaged)
		— assumes steady winds — difficult to adapt for heterogeneous and dispersive emission sources (more suited to singular point emission sources)

*In the context of UAV-based measurements.

UAVs provide a highly versatile platform, uniquely suited for high-density spatial mapping of methane plumes, and quantifying methane emissions at local scales. Their use could easily be incorporated into policy and regulation concerning monitoring and quantification of methane emissions from polluting industries [53] and also offer potential applications in hazard assessment (natural gas leaks), and extension to other emissions of interest, such as air quality pollutants.

## Data accessibility

This article has no additional data.
